# Early Referral for Breast-Cancer-Related Lymphedema: Do We Follow the Evidence? A Two-Year Prospective Multicenter Cohort Study

**DOI:** 10.3390/cancers14236016

**Published:** 2022-12-06

**Authors:** Ad A. Hendrickx, Saskia W. Küthe, Cees P. van der Schans, Wim P. Krijnen, Chantal M. Mouës-Vink, Robert J. Damstra

**Affiliations:** 1 Center of Expertise for Lymphovascular Medicine, Nij Smellinghe Hospital, Compagnonsplein 1, 9202 NN Drachten, The Netherlands; 2 Research Group Healthy Ageing Allied Health Care and Nursing, Hanze University of Applied Sciences, 9747 AS Groningen, The Netherlands; 3 Department of Health Psychology, University Medical Center Groningen, University of Groningen, 9712 CP Groningen, The Netherlands; 4 Department of Plastic, Reconstructive and Hand Surgery, Medical Center Leeuwarden, 8934 AD Leeuwarden, The Netherlands; 5 Department of Rehabilitation Medicine, University Medical Center Groningen, University of Groningen, 9712 CP Groningen, The Netherlands

**Keywords:** breast cancer, secondary lymphedema, early detection, referral, therapy, watchful waiting, cohort study, 2-years follow-up

## Abstract

**Simple Summary:**

The early detection of breast-cancer-related lymphedema and referral for therapy can reduce lymphedema-related problems. We aimed to determine whether the early detection of lymphedema and referral for treatment is adequate. Women treated for breast cancer were followed up within a standard protocol for two years with several measurements, including arm volumes. A 5% or greater Relative Volume Change was used to diagnose for lymphedema and as an indication for therapy referral. Among the patients with early signs of lymphedema, 83% of them were not referred for therapy. This remained consistent over a 2-year follow-up period. Additionally, we noticed a significant improvement of the mean Relative Volume Change at 24 months within this group. We concluded that waiting with a therapy referral and carefully monitoring if symptoms change may represent an appropriate choice when lymphedema is detected within the first year post-surgery.

**Abstract:**

The early detection of breast-cancer-related lymphedema and referral for therapy has the potential to reduce lymphedema-related morbidity. Although research shows the benefits, a gap is observed between evidence and daily practice. We aimed to determine whether the early detection of lymphedema and referral for treatment is adequate following the current guidelines. Women with primary breast cancer treated with breast-conserving therapy or ablative treatment were included. Demographic-, general health-, tumor-, and treatment-related data were recorded. Bilateral arm volume measurements were performed preoperatively and 3, 6, 12, and 24 months post-surgery. A 5% or greater Relative Volume Change was considered the cutoff point for lymphedema and as an indication for therapy referral. After 24 months post-surgery, the main outcomes show that among the patients with early signs of lymphedema, based on a Relative Volume Change ≥5%, a nonreferral for therapy was noted in 83%. Additionally, we observed a significant improvement of the mean Relative Volume Change at 24 months within this group, which might implicate that nonreferral was an adequate choice and that watchful waiting is appropriate when lymphedema is detected within the first year post-surgery.

## 1. Introduction

Lymphedema is one of the most common and well-known complications of breast cancer treatment. It is estimated that over one in five women will develop lymphedema (LE), potentially resulting in substantial physical, functional, and psychosocial burdens, along with financial burdens for patients and the health care system [1,2,3,4]. The early detection of breast-cancer-related lymphedema (BCRL) and referral for therapy has the potential to reduce lymphedema-related morbidity [5,6,7,8].

Breast-cancer-related lymphedema is a chronic condition that involves swelling of the arm and represents an imbalance between the rate of interstitial fluid generation (lymphatic load) and the lymphatic transport capacity. Breast cancer in general and specific components of its treatment can trigger this imbalance [9,10]. Well established risk-factors for the development of breast-cancer-related lymphedema include axillar lymphnode dissection (ALND), regional lymphnode radiation (RLNR), and high Body Mass Index (BMI) at the time of diagnosis [11]. Two phases in the development of LE are recognized: (I) a subclinical phase that involves fluid accumulation in the interstitium without clinical symptoms and (II) a phase of persistent LE with chronic inflammation that causes depositions of fatty tissue and fibrosis. Both stages reflect a compromised lymphatic system. The subclinical phase is generally reversible with conservative therapy, using treatment modalities such as patient education, self-management, and compression therapy. Conservative measures in the second phase will improve swelling in some patients, but the deposition of fatty tissue and fibrosis is generally irreversible. Therefore, the early detection and treatment of LE is important to prevent irreversible skin changes and will save intensive and expensive treatments [5,12,13,14,15]. 

Prospective surveillance programs aim to detect LE in an early phase. These programs should include objective measurement tools, standardized protocols, a pre-operative baseline assessment, and regular postoperative measurements to detect changes in the circumference of the upper extremities and weight changes [11,16,17]. Because most patients seem to present LE within the first two years after breast cancer surgery, more frequent surveillance during this time (e.g., once every 3–6 months) seems reasonable [1]. A cutoff point of 5% RVC for close monitoring or intervention was described by Specht et al. and is used in the Dutch guideline for lymphedema as well [13,18,19,20].

Although research shows the benefits of the early detection, referral, and treatment of BCRL, there is a gap between the evidence and daily practice [5]. Poor awareness of LE results in treatment delay and patients who lack the knowledge and skills to deal adequately with BCRL [21,22,23,24,25]. Alvarado et al. reported a difference between health care professionals regarding the risks they attribute to breast-cancer-related late effects such as LE and the indication for interventions such as patient education, more intensive screening, preventive measurements, and referral for therapy. Although relatively small, these differences suggest that late effect-related care among breast cancer patients varies and suggests that a substantial amount of patients do not receive appropriate treatment [26].

In this prospective cohort study, we aimed to determine whether the early detection and referral for lymphedema treatment is adequate in daily practice following the current guidelines. 

## 2. Methods

### 2.1. Study Design and Setting

This study used data from the Lymphedema Study Friesland (LOF), a prospective multicenter observational cohort study. The study was performed at the Medical Centre Leeuwarden and Nij Smellinghe Hospital in Drachten, The Netherlands. The study was conducted in accordance with the Declaration of Helsinki and approved by the local Medical Ethical Committees in Leeuwarden (number RTPO 974a) and in Drachten (number NH/JF/ID 1225). Patient enrollment started in December 2016 and closed in December 2019. Measurements were performed in a standardized manner by nurses specialized in oncology. A Case Report Form was developed to collect and store all data. 

### 2.2. Participants

Patients with a confirmed diagnosis of primary breast cancer scheduled for curative treatment were included after oral and written informed consent was provided to the department. Women treated with breast-conserving therapy or ablative treatment, with sentinel node dissection or axillar lymphnode dissection and with or without reconstructive surgery were included.

Patients with a history of prior treatment for breast cancer, preexisting lymphedema, preventive ablative treatment, and patients without axillar intervention (DCIS level 1 and partly DCIS level 2) were excluded. Patients who underwent bilateral breast cancer surgery were excluded from the analysis. Informed consent was obtained from all subjects involved in the study.

### 2.3. Study Outcomes

Demographic- and general health-related data were recorded prior to surgery, i.e., age, smoking, American Society of Anesthesiologists classification (ASA), body mass index (BMI), side of operation, and hand dominance. Tumor- and treatment-related data were recorded as soon as they were available during the diagnostic and treatment processes. Tumor-related data included tumor stage, the number of lymph nodes removed, and the number of positive lymph nodes. Treatment-related data included information on breast conserving therapy or mastectomy, sentinel node procedure or axillary lymph node dissection, neoadjuvant and adjuvant chemotherapy, hormonal therapy, and radiotherapy (including location) 

### 2.4. Arm Volume Measurement

Bilateral arm volume measurements were performed preoperatively and 3, 6, 12, and 24 months post-surgery. Arm volume measurements were obtained from both arms using the method of “Inverse Water Volumetry”, which is the gold standard for arm volume measurements [27,28]. To evaluate the arm volume changes over time, the formula described by Ancukiewicz was used [29]. This relative volume change (RVC) equation takes into account preoperative asymmetry between the arms and incorporates contralateral arm volume to account for changes in arm size caused by factors unrelated to lymphedema, such as weight gain. The formula is RVC = (A2U1)/(U2A1) − 1, where A1 and A2 are arm volumes on the side of the treated breast at two different time points, and U1 and U2 are volumes on the contralateral side. Patients who underwent bilateral breast cancer surgery were excluded from the analysis given that the RVC formula cannot be used for patients at risk for bilateral lymphedema. Based on the percentage values obtained from the RVC equation, the total group of patients was divided into two categories. The first category had an RVC < 5%, and the second category had an RVC ≥ 5%.

### 2.5. Early Therapy Referral

A 5% or greater volume increase (relative volume change) of the arm at the side of the surgery was considered the cutoff point for the diagnosis of LE and was considered an indication for therapy referral [18,20]. Therapy referral exclusively based on LE of the arm was recorded at 3, 6, 12, and 24 months in the Case Report Form and was categorized as follows: (0) no referral for therapy and (1) referral for therapy. At each of these time points, the incidence was scored for newly diagnosed patients with LE.

### 2.6. Self-Reported Signs and Symptoms

The medical records of the newly diagnosed LE patients were assessed for the presence of any self-reported signs and symptoms of LE (i.e., swelling, heaviness, tightness, stiffness, pain, sensory disturbance, and functional changes of the arm at the side of surgery) at the time point when the RVC increased by 5% or greater. The two first authors checked the files independently, argued the results, and resolved the differences by consensus.

### 2.7. Statistical Analyses

SPSS (IBM SPSS Statistics for Apple, Version 28.0.1.0, IBM Corp, 2021, Amsterdam, Netherlands) was used to generate the frequency tables, the proportion estimates, and crosstabulation, and to perform the paired-sample *t* tests. The chi-squared test and confidence intervals for the proportions of the RVC categories were calculated with the programming language R version 4.2.1 for statistical computing. Frequency tables were used to describe the demographic-, general health-, tumor-, and treatment-related characteristics of the study population. The proportions of patients with an RVC < 5% and with an RVC ≥ 5% were calculated, and these values were reported along with confidence intervals, mean RVCs, and standard deviations. Crosstabs were used to compute the number of patients referred and not referred for therapy in relation to the diagnosis of LE based on the RVC categories. A chi-squared test was used to test the hypothesis that there was no change in time since surgery for patients “not referred for therapy”, given a RVC of 5% or more was performed. Paired-sample *t* tests were conducted to compare the mean RVC of the newly diagnosed LE patients at 3, 6, and 12 months, with the mean RVC at 24 months. A *p* value of 0.05 or less was considered statistically significant.

## 3. Results

**Figure 1:**
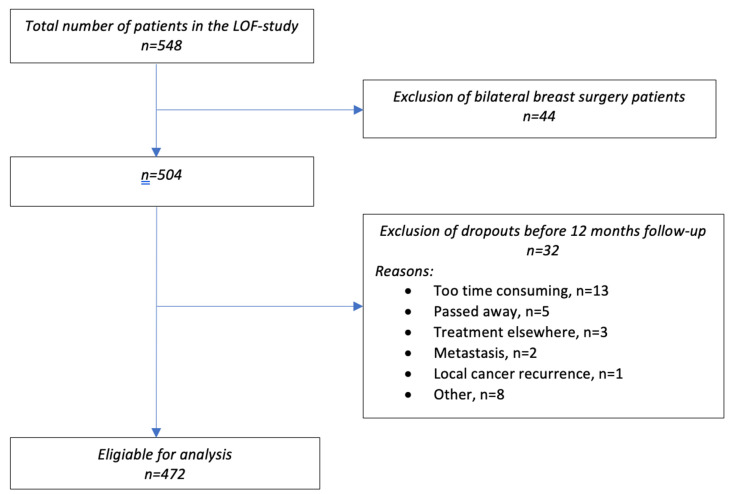
Flow diagram of eligible patients and those ultimately included in the analysis.

In total, 548 patients were included in the LOF study. After the exclusion of bilateral surgery patients and dropouts, data from 472 patients were included in the analyses. Figure 1 presents a flow diagram of the study.

### 3.1. Patient Characteristics

Table 1 shows the most relevant data regarding the demographic-, general health-, tumor-, and treatment-related characteristics of the study population.

### 3.2. Relative Volume Change Data

Table 2 shows the proportions of all patients based on RVC category, including non-LE with an RVC < 5% and LE with an RVC ≥ 5%, at 3, 6, 12, and 24 months.

### 3.3. Therapy Referral

Table 3 shows the number and percentages of patients referred and not referred for therapy in relation to the cutoff point of 5% or greater RVC. The number of newly diagnosed LE patients was 43 at 3 months, 19 at 6 months, 14 at 12 months, and 12 at 24 months.

The most interesting findings in Table 3 reflect the inappropriate choices made for specific groups: (I) the group with a diagnosis of LE (based on the RVC ≥ 5%) without a referral for therapy (this group is of upmost importance and directly related to our research question) and (II) the group without a diagnosis of LE (based on the RVC < 5%), but still referred for therapy. In the group with LE at 3 months, 35 patients (81.4%) were inappropriately not referred. Eight patients (18.6%) were properly diagnosed and referred for therapy. At 6, 12, and 24 months, the percentages of patients inappropriately not referred were 89.5%, 78.6%, and 83.3%, respectively. A chi-squared test on the frequencies of patients inappropriately not receiving a referral for therapy over the four time points since surgery shows no significant difference in the proportions (X-squared = 0.87, df = 3, *p* value = 0.84). The mean rate of nonreferral since surgery was 83% (95% CI, 0.73, 0.90).

### 3.4. Self-Reported Signs and Symptoms

Table 4 shows the number and percentages of newly diagnosed LE patients with self-reported signs and symptoms at different time points.

### 3.5. Assessment of the Mean RVC in Time within the LE Group

Table 5 shows the mean RVC data of newly diagnosed LE patients with and without a referral for therapy at 3, 6, and 12 months compared with the mean RVC at 24 months.

A significant mean decrease in RVC is observed in the group of newly diagnosed LE patients without a referral for therapy at all time points compared to 24 months. The group of patients who did receive a referral showed no significant change in RVC at all time intervals based on paired *t* tests.

## 4. Discussion

### 4.1. Key Findings

Our data show that 9.5% of the patients developed LE 3 months post-surgery, increasing to 10.5% at 2 years, using the cut-off point of 5% or greater RVC for the diagnosis. A minority of the newly diagnosed patients with LE also reported signs and symptoms in the first year post-surgery: 25.6% at 3 months, 26.3 % at 6 months, and 28.6% at 12 months, respectively. This percentage increased to 58.3% at 2 years. Among the patients with LE, an inappropriate nonreferral for therapy was noted in 83%. This value remained consistent over a 2-year follow-up period. At the same time, we observed a significant improvement of the mean RVC at 24 months in this group of LE patients without a referral for therapy, indicating that spontaneous improvement may occur. 

### 4.2. Consideration of Possible Mechanisms and Explanations

The current guidelines for lymphedema recommend referring patients for therapy in case of an RVC of ≥5%. If referral would strictly depend on the RVC value, without including any other (clinical) aspects or patient preferences, our results would show that these guidelines are not followed in the majority of the cases, since only 18.6, 10.5, 21.4, and 16.7 % of the patients with an RVC ≥ 5% were referred for therapy at 3, 6, 12, and 24 months post-surgery, respectively. An important question related to guideline adherence is whether deviations are intentional and supported by valid reasons or not [30]. In our case, the specialized oncological nurses may have considered, in dialogue with the patient, that “watchful waiting” is appropriate. In these cases, the first step is to provide additional information about LE to the patient and to schedule more frequent check-ups for clinical signs of LE and extra volume measurements. This stepped care model is supported by our study as: (I) there is an improvement in the mean RVC at 24 months compared with 3, 6, and 12 months, demonstrating that spontaneous resolution is possible; and (II) only a small percentage of the patients reported clinical signs and symptoms at 3, 6, and 12 months. However, at two years post-surgery, 58.3% of the newly diagnosed patients also reported signs and symptoms related to LE, which implicates that therapy could have been beneficial. Nevertheless, only 28.6% of them were referred. Additionally, if LE occurs two years after surgery, the chances for a spontaneous improvement are expected to be smaller, considering the time interval since surgery and the progressive nature of the disease [31,32]. In conclusion, the detection of LE in the first year after surgery seems adequate and the substantial number of patients with spontaneous recovery of the RVC supports the decision not to refer in some cases, and, therefore, supports the deviation from the guidelines. Although the group of patients inappropriately referred for therapy without a positive diagnosis of LE is small in size, they are a burden for health care costs. We will not further discuss this group, as it is beyond the scope of our research question. 

### 4.3. Relevant Findings from Other Published Studies

The observation in our group of newly diagnosed LE patients at 3, 6, and 12 months showing a significant improvement of the mean RVC at 24 months without a referral for therapy is consistent with research outcomes by Kilbreath et al. [33]. Using bioimpedance spectroscopy to detect LE, they found that swelling in the first year following surgery is likely to be transient. Other studies showed that LE might resolve with or without treatment in some patients, at least during the first months after surgery [2,34]. Additionally, research outcomes by Li Zou showed that LE can be accurately diagnosed only 1 month post-surgery, and Keeley demonstrated a relative volume increase of 5% or more at 1 month post-surgery to be a strong predictor for LE at 36 months [12,35]. 

Specht et al. assessed the optimal threshold for intervention in a group of 1173 patients treated for breast cancer and concluded that patients with a relative arm volume increase of 3% to <5% occurring later than 3 months after surgery did not have a statistically significant increase in the risk of progression to 10%. These researchers suggest the use of a 5 to <10% threshold for close monitoring or intervention in combination with risk stratification based on the presence of additional risk factors for the deterioration of LE [19]. 

Conservative treatment of more advanced stages of LE consists of Complex Decongestive Therapy (CDT), with elements such as manual lymph drainage, compression bandages, skincare, and exercise. In relation to the current debate about the relevance and contribution of the different treatment modalities within the CDT, a recent review of de Site et al. underlines the importance of compression therapy as the cornerstone of the treatment and advocates the use of a multidisciplinary integrated approach emphasizing exercise therapy, self-management, and lifestyle [36].

Screening for subclinical lymphoedema to allow early intervention is internationally recommended, although the evidence is inconclusive and limited due to small sample sizes, the lack of a control group, and limited follow-up data [12]. Stout et al. were the first to demonstrate the benefits of early detection and used compression garments for a period of 4 weeks to prevent progression [37]. Similar results were found by Whitworth et al. [38,39]. Koelmeyer et al. demonstrated a low rate of progression from subclinical LE to LE, with an RVC ≥ 10% based on early recognition and interventions, including education, self-monitoring, and compression garments [5]. Finally, a systematic review by Jeffs et al. on the clinical effectiveness of decongestive treatments in early BCRL found weak evidence (grade B) for the impact of decongestive lymphedema treatment. In this study, early LE was defined as BRCL symptoms with less than 12 months of duration. These researchers could not identify the optimal treatment components to reduce excess arm volume or determine the optimal duration of the treatment [40]. 

### 4.4. Potential Implications of Our Results for Daily Practice

The period of swelling can be considered as a first signal of a compromised balance between the lymphatic load and the lymphatic transport capacity, and as an indication for the risk for recurrence. However, this period is also considered as transient. From this perspective, our results suggest that “watchful waiting” and starting with additional information and extra monitoring (in our situation, performed by the oncological nurses) might represent a good treatment option for the patients newly diagnosed in the first year post-surgery, with a cut-off point for LE of RVC ≥ 5%. The limited evidence regarding the treatment after early detection supports this choice for watchful waiting. Choosing for watchful waiting could also reduce health care costs. Given that we did not collect data regarding the additional interventions performed by the nurses, we cannot draw conclusions about the impact of these interventions. It is reasonable to expect that these interventions were performed adequately and played a positive role. In addition, the nurses play a central role during the first years post-surgery by guiding these patients on a regular basis during oncological follow-up. As qualified “case managers”, they are aware of the undesirable side effects of cancer treatment and are equipped to inform and instruct patients about LE. Another relevant aspect to note is that patients can easily approach them when they have new questions, notice an increase in volume, or experience other symptoms. More generally, we believe that the growing awareness in cancer-survivorship care regarding exercise, as well as for the risks of obesity, will have played a positive role in the maintenance of the balance between the lymphatic load and the lymphatic transport capacity.

Based on our results, questions could be raised about the current guidelines. The cutoff point of a 5% or greater increase in RVC could be too low given the number of patients doing well without a referral for therapy. If these patients had been referred for therapy, over-treatment could have occurred. However, considering the importance of early detection, the discussion is probably better focused on the question “after early detection, what is next?”. In addition to watchful waiting with the health care professional in the lead in the first period after the detection of LE, patients should be encouraged to improve their self-management. Regarding the risks of recurrence of LE, patients should be educated about LE in general and more specifically about the risks for progression, and taught how to monitor themselves and when to seek professional support.

### 4.5. Strength, Limitations, and Recommendations

Our study has several strengths. First, the observational design created an optimal reflection on daily practice. Second, the follow up of two years allowed us to evaluate arm volume changes in probably the most relevant period in the development of BCRL. Third, the data collection protocol assured reliable and accurate results. Our study also has some limitations. The first limitation is the lack of insight in the arguments and considerations used by the oncological nurses in their decision-making process for therapy referral. A second limitation concerns the lack of data regarding the additional interventions performed by the oncological nurses. A third limitation concerns the use of data concerning the self-reported signs and symptoms derived from the medical records, as this is associated with risks of incomplete files and interpretation errors. Further comprehensive research on LE detection and referral for therapy will help us to better understand and optimize the decision-making process. Research regarding the amount and content of the interventions performed by the nursing staff and by the patients themselves in the early phases of LE will help us to better understand the best treatment methods to employ when early symptoms of LE are detected. An on-site observational or participative study design could be a good choice for these purposes.

### 4.6. Conclusions

Within the growing body of evidence for the early detection of BCRL, our study highlights the need to focus on the step after the detection of early signs of LE. The results show that RVC outcomes should be combined with other clinical features, risk factors, and patient preferences in order to find the most optimal and individually tailored intervention for the patient. 

Our data support the use of watchful waiting as an appropriate choice when LE is detected within the first year post-surgery, using a threshold of RVC ≥ 5%. 

## Figures and Tables

**Table 1:** Patient characteristics.

Characteristics	Mean ± SD	Number of Patients	Frequency %
**Demographic & general health related**			
Age (years)	59.2 ± 10.4		
Smoking			
- *Current smoker*		60	12.7
- *Never*		208	44.2
- *Ex-smoker*		203	43.1
ASA classification			
- *ASA 1*		85	18.1
- *ASA 2*		294	62.6
- *ASA 3*		88	18.7
- *ASA 4*		3	0.6
BMI preoperative	27.3 ± 5.1		
- *Cat 1, ≤25*		162	34.5
- *Cat 2, >25–≤30*		198	42.1
- *Cat 3, >30*		110	23.4
Dominant side surgery		226	49.9
**Tumor related**			
Tumor stage			
- *0*		3	0.9
- *1*		72	21.5
- *2*		167	49.9
- *3*		93	27.8
Number of removed lymphnodes			
- *<5*		428	90.7
- *≥5*		44	9.3
Total number of positive nodes ≥1		141	29.9
**Treatment related**			
Breast conserving therapy (BCT)		361	76.5
Mastectomy		120	25.4
Sentinel node procedure		450	95.3
Axillar lymph node dissection		34	7.2
Neo-adjuvant chemotherapy		111	23.5
Adjuvant chemotherapy		107	22.7
Hormonal therapy		287	60.8
Radiation		400	84.7
- *Including the axilla*		132	33.0

Valid percentages are used to correct for missing data.

**Table 2:** RVC development over time of all patients based on RVC category.

Follow-Up	RVC < 5%		RVC ≥ 5%	
	Proportion	95% CI	Proportion	95% CI
3 months (*n* = 455)	90.5	87.4, 93.0	9.5	6.9, 12.6
6 months (*n* = 449)	91.3	88.2, 93.7	8.7	6.3, 11.8
12 months (*n* = 448)	91.3	88.2, 93.7	8.7	6.3, 11.8
24 months (*n* = 437)	89.5	86.1, 92.1	10.5	7.9, 13.9

RVC = Relative Volume Change. Valid percentages are used to correct for missing data.

**Table 3:** Numbers and percentages of newly diagnosed patients referred and not referred for therapy, in relation to the diagnosis LE.

Therapy Referral	RVC < 5%	RVC ≥ 5%
	*n* (%)	*n* (%)
**3 months**		
No	411 (99.8)	35 (81.4)
Yes	1 (0.2)	8 (18.6)
**6 months**		
No	371 (97.9)	17 (89.5)
Yes	8 (2.1)	2 (10.5)
**12 months**		
No	340 (97.1)	11 (78.6)
Yes	10 (2.9)	3 (21.4)
**24 months**		
No	314 (98.4)	10 (83.3)
Yes	5 (1.6)	2 (16.7)

RVC = Relative Volume Change.

**Table 4:** Percentages of newly diagnosed LE patients with self-reported signs and symptoms.

Follow-Up	% (95% CI)
**3 months (*n* = 43)**	25.6 (14.0, 41.5)
Not referred	45.5 (18.1, 75.4)
Referred	54.5 (24.6, 81.9)
**6 months (*n* = 19)**	26.3 (10.1, 51.4)
Not referred	60.0 (17.0, 92.7)
Referred	40.0 (7.3, 83.0)
**12 months (*n* = 14)**	28.6 (9.6, 58.0)
Not referred	25.0 (1.3, 78.1)
Referred	75.0 (21.9, 98.7)
**24 months (*n* = 12)**	58.3 (28.6, 83.5)
Not referred	71.4 (30.3, 94.9)
Referred	28.6 (5.1, 69.7)

**Table 5:** Group level data of the mean RVC of the LE patients, over three time points (the results are obtained by paired t testing).

Follow Up.	Mean (SD) RVC %	T24, Mean (SD) RVC %	Mean Difference (%, SD)	*p* Value
**No treatment referral**				
T3 (*n* = 33)	8.7 (4.6)	5.3 4.0)	−3.4 (5.8)	0.002 *
T6 (*n* = 16)	9.9 (11.6)	3.6 (3.2)	−6.3 (11.6)	0.045 *
T12 (*n* = 11)	7.8 (2.6)	2.4 (4.3)	−5.4 (5.7)	0.011 *
**Treatment referral**				
T3 (*n* = 7)	10.8 (5.9)	15.9 (16.5)	5.1 (17.2)	0.467
T6 (*n* = 2)	8.2 (1.2)	9.2 (10.3)	1.0 (9.1)	0.899
T12 (*n* = 3)	9.4 (2.2)	6.6 (3.4)	−2.8 (2.2)	0.163

RVC= Relative Volume Change. Mean RVC’s at 3, 6 and 12 months post-op are compared with the mean RVC at 24 months. * Significant at *p* < 0.05.

## Data Availability

The data are available from the corresponding author upon reasonable request.
